# Equitable Healthcare Access for Type 2 Diabetes Patients Under a Low-Income Group Health Care Scheme: A Sustainable Development Goal Perspective

**DOI:** 10.3390/ijerph22060817

**Published:** 2025-05-22

**Authors:** Sin Wei Tey, Kingston Rajiah, Mari Kannan Maharajan, Norasila Binti Zakaria, Nor Haslinda Binti Ishak

**Affiliations:** 1 School of Postgraduate Studies, International Medical University, Kuala Lumpur 57200, Malaysia; teysinwei@gmail.com; 2 School of Pharmacy and Pharmaceutical Sciences, Ulster University, Coleraine BTS2 1SA, UK; 3 School of Pharmacy, University of Nottingham, Semenyih 43500, Malaysia; marikannan.maharajan@nottingham.edu.my; 4 Klinik Kesihatan Ayer Molek, Melaka Tengah Health District Office, Melaka 75460, Malaysia; asilanor@gmail.com; 5 Klinik Kesihatan Batu Berendam, Melaka Tengah Health District Office, Melaka 75350, Malaysia; drnorhaslinda@moh.gov.my

**Keywords:** access to care, diabetes, primary care, health equity, Malaysia

## Abstract

Objective: The purpose of this study is to explore the factors influencing access to healthcare services among Type 2 Diabetes Mellitus (T2DM) patients enrolled in the PEKA B40 programme at a public health clinic in Melaka, Malaysia. It aims to examine how key dimensions—availability, accessibility, accommodation, and acceptance—affect patients’ experiences and the utilisation of diabetes-related healthcare services. This study also seeks to identify gaps and challenges within the current healthcare delivery system, particularly for low-income populations, to inform strategies for improving equitable and sustainable access to care. Methods: This study was conducted in a public health clinic in Melaka, Malaysia. Purposive sampling was used among T2DM patients under the “Health Care Scheme for Group B40” programme in a public health clinic in Melaka, Malaysia. The study included participants with at least a 6-month history of T2DM to ensure substantial experience in accessing healthcare services. Results: Fifteen patients participated in this study. Elderly individuals, retirees, and those with average incomes demonstrated higher healthcare service utilisation. Ethnic diversity was crucial, revealing its impact on health behaviours and healthcare-seeking patterns. Primary or secondary education levels among participants highlighted the necessity for targeted health literacy efforts. Conclusions: This study highlighted notable awareness and satisfaction among patients concerning the availability, accessibility, and accommodation of services, particularly emphasising the importance of geographical proximity in healthcare services. However, challenges faced by elderly individuals in accessing social support are also highlighted. The potential of enhancing the amenities of healthcare facilities to improve patient experiences is also reflected in our results. These insights provide evidence for the effectiveness of the Malaysian healthcare system in catering to a diverse demographic and can also be helpful in refining healthcare strategies and further optimising patient-centred care in Malaysia.

## 1. Introduction

Access to care is pivotal in healthcare and encompasses five key dimensions: affordability, availability, accessibility, accommodation, and acceptability [1]. These dimensions ensure that healthcare services are economically feasible [2], adequately resourced with facilities, professionals, and essential medical supplies [3], geographically accessible [4], patient-centric [5] and culturally sensitive [6]. Understanding these dimensions is crucial in multiracial and multicultural societies and will be helpful in a healthcare system that is inclusive, equitable, and responsive to diverse needs.

As of 2019, approximately 3.9 million adults in Malaysia are living with diabetes, with the majority presenting with Type 2 Diabetes Mellitus (T2DM). This represents a significant public health concern, as the prevalence of diabetes among adults aged 18 and above increased from 11.2% in 2011 to 18.3% in 2019 [7]. Regarding the PEKA B40 programme, which aims to provide health services to the lower 40% income group (B40), recent data indicate that only about 10% of eligible individuals have utilised the free health screenings offered. Specifically, out of the six million Malaysians eligible under this scheme, only approximately 600,000 have availed themselves of its benefits over the past three years [8]. These statistics highlight the need for enhanced awareness and utilisation of healthcare services among T2DM patients, particularly within the B40 group, to ensure effective management and better health outcomes.

In Malaysia, despite the widespread availability of highly subsidised government healthcare facilities [9], disparities in access and inequitable health outcomes persist, particularly among socially disadvantaged groups [10], including the elderly and low-income individuals [11]. Limited access to public clinics for these groups is influenced by factors such as age, education, income and the need for care among the elderly [12]. However, detailed insights into how these factors affect the access and utilisation of healthcare services remain unexplored.

In 2019, the Malaysian government introduced the Peduli Kesihatan scheme (PeKA B40), which means “Health Care Scheme for Group B40” for the lower 40% income group to improve access to care for Non-Communicable Diseases (NCDs) [13]. The eligibility of PeKa B40 is automatic based on the set criteria, namely recipients of Sumbangan Tunai Rahmah (STR), which means “Compassion Cash Contribution”, and their registered spouses who are aged 40 years and above. No separate registration is required to join PeKa B40 [14]. This scheme covers a range of benefits, including NCD screening, financial support for medical treatment, and transportation for hospital care [15], and hence, it addresses the affordability of beneficiaries. However, the dimensions of the availability, accessibility, accommodation, and acceptability of PeKa B40 beneficiaries have not been explored. Very few studies have been reported on PeKa B40 beneficiaries. Studies have stated the necessity of understanding the factors that affect the healthcare access of patients with NCDs [16,17].

Globally, the socio-economic inequalities observed in the progression of NCDs and the risk of diabetes complications are a concern [18]. In Malaysia, according to the World Health Organisation (WHO), Type 2 Diabetes Mellitus (T2DM) is one of the most common NCDs [19]. The global issue is reflected in Malaysia, as the socio-economic inequalities among the B40 group result in varied access to and utilisation of healthcare services for NCDs, especially diabetes [9,10,11,12]. Understanding the gaps in accessing and receiving appropriate healthcare is crucial for shaping policies and delivering more equitable healthcare services. Hence, this study focused on T2DM patients under the PeKA B40 programme to explore their experiences and challenges in accessing care.

## 2. Materials and Methods

### 2.1. Study Design and Sample

This study was conducted in a public health clinic in Melaka, Malaysia. Purposive sampling was used among T2DM patients under the PeKa B40 programme in Klinik Kesihatan (KK) in Melaka, Malaysia. It is a public health clinic that serves a significant population within the Melaka state and is actively engaged in the PeKa B40 programme. However, the study did not include specific data on the clinic’s size or the total number of registered diabetes patients.

The study included participants with at least a 6-month history of T2DM to ensure substantial experience in accessing healthcare services. Participants were identified by confirming their eligibility using the clinic’s PeKa B40 account and were approached during their visit to the pharmacy department of the clinic. The researchers aimed to achieve diversity across race, gender, and age groups. However, participant demographic information was not accessed in advance. Instead, potential participants were approached during their routine visits to the pharmacy department, and eligibility was assessed through their PeKa B40 registration status and confirmation of Type 2 Diabetes Mellitus (T2DM) history via the Pharmacy Information System. Diversity was sought progressively during the recruitment process based on observed characteristics and self-disclosed information during initial screening. While the intent was to ensure demographic variation, the study did not predefine age categories nor stratify recruitment targets by demographic quotas. Each participant who agreed to participate in the study was provided with an overview of the study, which included the study’s purpose, potential risks and benefits, and the time it would take. Participation was voluntary, and participants were allowed to withdraw at any time during the interview. Written informed consent was obtained from all participants. Sampling was performed until saturation was reached. The saturation point was determined by analysing the collected data, which indicated that further collection of data may not bring any new themes. In other words, the researchers reached a point where no new themes were generated from the data collected.

### 2.2. Interview Guide

A semi-structured interview guide was developed by reviewing the literature on access to care [20,21,22]. (Refer to Supplementary Materials). Open-ended questions were used to provide interviewees with a full opportunity to convey their opinions and to obtain a greater understanding of issues. A pilot interview was conducted with two participants. The questions were rephrased based on the pilot interview. The data collected during the pilot interviews were not included in the results.

### 2.3. Data Collection

In-depth interviews were conducted. The interview duration was between 30 and 45 min. All interviews were audio recorded. The interview recordings were subsequently transcribed verbatim by the researcher. The accuracy of all the transcripts was checked by two researchers. The transcripts were subsequently returned to the participants for comments and corrections. The final transcripts were stored in password-protected Microsoft Office Word documents.

### 2.4. Data Analysis

The transcribed data were coded and analysed using thematic analysis [23], with a focus on identifying emergent themes. Initially, the data were examined for common patterns and sorted into categories based on similar trends. These categories were then coded and labelled, with notes recorded on key ideas that arose during the process. Thematic analysis was conducted following a structured approach: the researchers first familiarised themselves with the data, then generated initial codes, followed by searching for relevant themes, reviewing and refining those themes, and finally defining and naming them. The final step involved producing a comprehensive report.

### 2.5. Data Trustworthiness

Rigour in the study was evaluated using the criteria of credibility, dependability, transferability, and confirmability. To ensure the trustworthiness of the research, the study adhered to the Standards for Reporting Qualitative Research (SRQR) guidelines. Credibility was established using open-ended questions to elicit authentic responses from participants, allowing for a deeper understanding of their experiences. The research team achieved consensus on coding through iterative discussions, and thematic findings were validated by participants to ensure an accurate representation of their perspectives. Dependability was demonstrated by aligning the findings with the relevant literature and established theories, thereby reinforcing the consistency and reliability of the results. The research process was conducted with a high level of transparency and traceability, including detailed documentation of data collection, analysis, and key decision-making steps. This also involved maintaining logs of significant decisions, reflective notes, and the secure storage of raw data, along with records of participant feedback and peer reviews. Transferability was enhanced by providing rich, detailed descriptions of participant characteristics, the data analysis process, and original excerpts from participant dialogue, allowing readers to determine the relevance of the findings to other contexts.

## 3. Results

### Participants’ Demographic Characteristics

Participants’ demographic characteristics are presented in Table 1. Fifteen in-depth interviews were conducted in total. The age of the participants ranged from 48 to 79 years, with most being older than 65 years. Nine of the participants were female. In terms of ethnicity, eight were Malay, four were Chinese, and three were Indian. In terms of education, seven received primary education and eight received secondary education. None of the participants were tertiary qualification holders. The history of T2DM among the participants ranged between 6 months and 20 years, with most of them having a diagnosis for more than 5 years. Eleven participants were either homemakers or retirees.

Table 2 lists the main themes, sub-themes and key findings.

- Main theme 1: Availability.
- Sub-theme 1.1: Utilisation of services available.

Participants were aware of the basic services that they routinely received for their diabetes management, such as doctor’s consultations, medication collection, and blood and urine tests. X-ray and fundoscopy services were familiar to most patients, except those who had received diabetes treatment for less than a year. However, physiotherapy and dietitian counselling were less known to diabetes patients, even for those who had been on regular follow-up for a few years.

- Sub-theme 1.2: Ease of service utilisation.

Participants were satisfied with the medical equipment and services available and their ease of utilisation. A wide range of service coverage, such as X-ray and fundoscopy and co-located with dental services, was the reason for patients’ satisfaction levels.

- Main theme 2: Accessibility.
- Sub-theme 2.1: Clinic in the vicinity.

Participants mentioned that the clinic is located only a short travel distance from their residence. None had transportation problems in accessing the clinic.

- Sub-theme 2.2: Mode of travel.

Some participants drove themselves to the clinic, and others relied on family members to fetch them to the clinic. None of them reported using public transport. Despite the majority of the participants having no barrier to physically reaching the clinic, a few described that they had some trouble finding people to visit the clinic. Some participants received help from others for monthly medication collection.

- Main theme 3: Accommodation.
- Sub-theme 3.1: Waiting time.

The waiting time mentioned by the participants is the time taken from registration to finish collecting medications. Participants were aware that they should wait to receive healthcare services. The waiting time in the clinic ranged from 30 min to 3 h. Most participants reported that a waiting time of 2 h was acceptable. Some participants complained about the waiting time when asked about the usefulness of services or problems encountered in utilising the services.

- Sub-theme 3.2: After-hours and walk-in service.

Participants mentioned that the clinic’s opening hours from 8 am to 5 pm during weekdays were not restricting them and did not have an impact on their healthcare accessibility. None of them were concerned about after-hours accessibility to primary care service during evenings, nights, weekends, or walk-in service. When asked about the need for healthcare after-hours, most participants mentioned that they used emergency services and self-medicated. Some mentioned that they have not encountered the need so far.

- Sub-theme 3.3: Frequency of follow-up.

Regular follow-up is part of diabetes care management. The interval of time between each follow-up is determined by the doctor according to the clinical status of the patient and the treatment regimen. Generally, the time intervals for adjusting the therapeutic plan and performing laboratory tests range from two weeks for patients who require close monitoring to six months for stable patients. Most participants were satisfied with the interval given for their follow-up. However, one participant had a different opinion.

- Sub-theme 3.4: Facilities and amenities.

The examples of facilities and amenities included were the adequacy of parking lots, toilets, chairs for sitting in, and the conditions of the waiting lounge and consultation rooms. Most participants were satisfied with the facilities and amenities. Suggestions were given by some participants to increase the number of toilets and parking lots.

- Main theme 4: Acceptance.
- Sub-theme 4.1: Patients’ acceptance of providers’ characteristics and vice versa.

Owing to the diversity of race, culture, language, and religion in Malaysia, it is important to gain access to healthcare services. None of the participants reported issues in acceptance regarding the characteristics of the providers and vice versa. None of the participants reported any age, gender, or ethnicity preference for receiving care.

- Sub-theme 4.2: Communication and interaction with healthcare providers.

Most participants reported good interaction and communication with the healthcare providers. They agreed that they received sufficient updates about their health from the doctors, and their questions were answered clearly by the doctors. Very few participants reported a language barrier as they were not well-versed in the Malay language. Translation assistance from others, including healthcare staff, patients or family members, was gained when asked about how communications were tackled. In addition, the suggestion to establish a multiethnic healthcare team in the clinic to overcome the language barrier problem was also given by these participants.

- Sub-theme 4.3: Patients’ Perceptions of the Staff.

None of the participants reported any concern about the staff. Participants commonly use the words “helpful”, “friendly”, and “polite” to describe the attitude of the staff.

## 4. Discussion

The principal findings shed light on several key aspects of healthcare delivery and patient experience within the context of diabetes management. While patients were generally aware of core services such as doctor consultations and medication collection, awareness varied for services like physiotherapy and dietitian counselling, suggesting potential areas for improvement in patient awareness. Satisfaction with available services and their ease of utilisation highlight the importance of comprehensive care provision, particularly with the inclusion of services like X-ray and fundoscopy alongside dental services. Clinic proximity was noted as favourable, although challenges in finding assistance for clinic visits or medication collection were reported, indicating potential barriers for certain patient groups. While waiting times at the clinic were generally acceptable, suggestions for improvements, such as extended hours or alternative service models, were noted. The satisfaction expressed regarding follow-up appointment frequency highlights effective collaboration between patients and providers, though personalised approaches might be warranted based on individual clinical needs. Suggestions for enhancing facilities and amenities, such as increasing toilets and parking lots, provide insights for optimising the clinic environment. The acceptance of healthcare providers’ characteristics across diverse demographics reflected positively on the inclusivity of care delivery. Strategies to address language barriers, such as translation assistance, were identified as important for effective communication and accessibility. Positive perceptions of clinic staff indicated a high standard of patient-centred care.

The strength of this study is its exploration of factors influencing healthcare access for T2DM patients in the PeKA B40 programme. By integrating a range of demographic, socioeconomic, and experiential factors, this study offers valuable insights to policymakers and practitioners. The inclusion of participants from diverse demographic backgrounds enhances the generalisability of findings and offers insights into different perspectives. Qualitative data capture experiences and interactions, providing rich insights for healthcare providers and policymakers. However, despite efforts to include diverse participants, the findings may not fully generalise to all diabetes patients as it has a single-site focus.

Inequity in diabetes care is a complex challenge that requires significant effort with a special focus on social and healthcare systems. Access to healthcare services is one of the effective management strategies in the management of T2DM. The findings revealed a complex interplay between factors such as availability, accessibility, accommodation, and acceptance and how they impact T2DM patients under the PeKA B40 programme in Melaka, Malaysia. In this study, participants were over 65 years of age and predominantly homemakers or retirees. This demographic trend aligns with previous reports indicating higher healthcare utilisation among elderly individuals living independently and those with average income levels [24]. This study included participants from various ethnicities, shedding light on their health behaviours, treatment preferences, and healthcare-seeking patterns. These health-seeking patterns are related to both people’s social values and culture [25]. Most participants had primary or secondary education, indicating the need for targeted health literacy initiatives. Limited educational attainment can adversely affect an individual’s ability to manage diabetes effectively, adhere to treatment regimens and access healthcare services [26]. The duration of T2DM among the majority of participants exceeded five years, which points to the challenges of long-term disease management. This finding emphasises the need for ongoing support, follow-up, and education in diabetes care. The occupation status of participants, predominantly homemakers or retirees, suggests unique challenges for these groups in accessibility, aligning with global observations regarding healthcare accessibility for unemployed individuals [27]. Our study revealed participants’ substantial awareness of the availability of diabetes management services, including doctor’s consultations, medication collection, and blood and urine tests. This awareness is likely a result of the Malaysian government’s focus on NCDs in lower-income groups [28]. However, a noteworthy observation is the limited knowledge of specialised services such as physiotherapy and dietitian counselling, irrespective of their regular follow-up for more than five years. Such gaps in awareness may arise from inadequate emphasis on holistic care or insufficient communication regarding these services. Participants expressed satisfaction with the medical equipment and services available for diabetes management, highlighting the user-friendly nature of the healthcare system. The integrated service approach used, which combines various medical services in one location, is particularly appreciated for its convenience. Geographical proximity and ease of transportation emerged as positive aspects of healthcare accessibility. The strategic location of clinics nearer to residential areas, a result of the Malaysian government’s efforts to minimise urban–rural healthcare disparities [29], facilitated easy access. However, challenges in social support and medication collection highlighted the importance of community networks and direct-to-consumer prescription drug advertising (DTCA) [30]. Recognising and addressing these challenges can contribute to a more comprehensive approach to healthcare accessibility for individuals with T2DM, ensuring that both geographical and social factors are considered in optimising patient-centred care. In terms of accommodation within the healthcare setting for individuals managing T2DM, mixed perspectives were seen. Although most participants found waiting times within 2 h acceptable, complaints appeared when assessing the overall usefulness of services. The clinic’s operating hours, from 8 am to 5 pm on weekdays, were generally perceived as non-restrictive, with participants expressing satisfaction with this timeframe. Interestingly, there were no concerns about after-hours primary care services or walk-in cases, with participants largely relying on emergency services and self-medication. This is mainly due to the Malaysian government’s free health services at private clinics through the Madani Medical Scheme for the B40 group. The free services offered in this scheme include consultation, check-ups, medicines, procedures, and referrals at private clinics, especially at night and on weekends [31]. The frequency of follow-up, a crucial aspect of diabetes care management, received positive feedback overall, with participants expressing satisfaction with the intervals determined by doctors. The results emphasise the importance of addressing waiting times, accommodating after-hours needs, and continuously enhancing facilities and amenities to optimise the overall patient experience for those managing T2DM. In this regard, the Malaysian government was also shown to focus on primary healthcare services and community-based services to improve the delivery of healthcare services through better stakeholder engagement. Facilities and amenities received positive feedback, but suggestions for infrastructure improvements and calls for the sustainability of the healthcare system were reported. In this regard, the Malaysian government is introducing the Malaysian National Health Policy (MNHP), which aligns with global goals advocated by the WHO. Particularly, the Health White Paper (HWP) prioritises population health and aims to ensure the sustainability of the healthcare system in Malaysia [32].

The findings of this study highlight the need for policy initiatives aimed at improving healthcare service delivery for diabetes patients in Malaysia. Policymakers should prioritise initiatives that enhance the accessibility and availability of essential services, particularly for marginalised populations. Strategies to address transportation challenges and extend clinic hours could improve access to care. Additionally, policies promoting cultural competence and language diversity within healthcare settings can help address communication barriers and improve patient–provider interactions. Healthcare practitioners can utilise the insights from this study to inform their practice and enhance patient-centred care. Improving communication strategies, such as providing translation assistance or establishing multiethnic healthcare teams, can facilitate better patient–provider interactions. Practitioners should also consider the diverse needs of diabetes patients and tailor services accordingly, including personalised follow-up schedules and culturally sensitive care approaches. Addressing facilities and amenities based on patient feedback can further enhance the overall patient experience. Future research should build upon the findings of this study to further advance our understanding of healthcare service utilisation among diabetes patients in Malaysia.

### Limitations

This study has several limitations that should be acknowledged. Firstly, it was conducted at a single public health clinic in Melaka, potentially limiting the generalisability of findings to other regions or healthcare settings. Detailed information about the clinic’s size and the total number of T2DM patients was not reported, which restricts the contextual interpretation of the results. Secondly, while purposive sampling aimed to include participants of different racial, gender, and age backgrounds, demographic data were not accessed in advance, and recruitment was based on convenience during routine pharmacy visits. This may have introduced selection bias and limited the diversity of the sample. Thirdly, age categories were not predefined or systematically reported, which constrains the analysis of age-specific access issues. Additionally, the exclusion of newly diagnosed T2DM patients (with less than 6 months since diagnosis) may have overlooked the unique access challenges faced by this group. Finally, the reliance on self-reported data may have introduced recall or social desirability bias, particularly in participants’ responses about service satisfaction and staff interactions.

## 5. Conclusions

This study aimed to understand the experiences and challenges of Type 2 Diabetes Mellitus patients under the PeKA B40 programme in accessing and receiving appropriate healthcare. The findings reveal several gaps and areas for improvement despite overall positive experiences. Most individuals were familiar with essential diabetes management services like doctor consultations, medication collection, and laboratory tests. However, there was limited awareness and utilisation of physiotherapy and dietitian counselling, even among long-term patients. This indicates a need for increased education and promotion of these critical services to enhance comprehensive diabetes management. Accessibility was generally satisfactory, with clinics located conveniently close to individuals’ residences and most patients having reliable transportation. Yet, some individuals faced challenges in arranging visits, relying on others for transportation and medication collection, highlighting the need for better support systems for those with limited personal mobility or social support. Participants appreciated the range of services offered and found the clinic environment and medical equipment satisfactory. However, waiting times varied significantly, with some finding longer wait times unacceptable. This suggests a need for improved clinic efficiency and possibly the introduction of appointment-based systems to reduce waiting times. The clinic’s operating hours were sufficient, with no significant demand for after-hours services, as participants resorted to emergency services or self-medication for urgent needs. Cultural and linguistic diversity did not significantly hinder access to care, with most participants reporting good communication with healthcare providers. However, a few faced language barriers, highlighting the importance of having a multiethnic healthcare team or translation services available.

## Figures and Tables

**Table 1:** Participants’ demographic characteristics.

Characteristics	Participants n = 15
Age in years	48–79
Gender	
Male	6
Female	9
Ethnicity	
Malay	8
Chinese	4
Indian	3
Education level	
Primary	7
Secondary	8
Tertiary	0
Marital status	
Married/living with partner	13
Unmarried/living alone	2
History of Type 2 Diabetes	6 months–20 years
Employment status	
Employed	4
Homemaker/retiree	11

**Table 2:** Themes, sub-themes, and key findings.

Main Themes	Sub-Themes	Key Findings
Availability	Utilisation of service available	Commonly known services: doctor’s consultation, medication collection, blood and urine test, X-ray, and fundoscopy services. Physiotherapy and dietitian counselling were less known to the participants.
	Ease of service utilisation	Participants were satisfied with the medical equipment, the wide range of service coverage, and their ease of utilisation.
Accessibility	Clinic in the vicinity	The clinic is located in the vicinity of participants’ residences. No transportation problem was reported.
	Mode of travel	Self-drive or rely on family members. Difficulty finding means to reach a clinic for those relied on family members.
Accommodation	Waiting time	Aware of the need to wait. The majority think the waiting time is acceptable; however, complaints still exist.
	After-hours and walk-in service	No difficulty in access. They use private clinics, hospital emergency department services, self-medicate or have not encountered the need so far.
	Frequency of follow-up	Mixed opinions regarding the interval of follow-up.
	Facilities and amenities	Participants are satisfied with the facilities and amenities.
Acceptance	Patients’ acceptance of providers characteristics and vice versa	No issues with acceptance, and care was delivered equally regardless of the characteristics of the providers and vice versa.
	Communication and interaction with healthcare providers	Good interaction and communication. Language barrier.
	Patients’ perceptions of the staff	Positive feedback on the attitude of the staff.

## Data Availability

The data presented in the study are stored securely at the School of Pharmacy, International Medical University. The investigators act as custodians for the data processed and generated by the study and they are also responsible for the access to any information included. Data are available upon request from the corresponding author. Due to privacy and institutional regulations, the data are not publicly accessible.

## Supplementary Materials

The following supporting information can be downloaded at https://www.mdpi.com/article/10.3390/ijerph22060817/s1. File S1: questionnaire.
