# Supplementary material for: Equitable Healthcare Access for Type 2 Diabetes Patients Under a Low-Income Group Health Care Scheme: A Sustainable Development Goal Perspective
Source: Int J Environ Res Public Health. 2025 May 22;22(6):817. doi: 10.3390/ijerph22060817 (PMC12193216; doi:10.3390/ijerph22060817)
Supplement: Supplementary file 1 [file ijerph-22-00817-s001.zip › ijerph-3542733-supplementary.pdf]

Availability and Use of Services

1. What types of services or resources are available to you at this clinic for managing your diabetes?

Probes: Doctor consultations, medications, blood/urine tests, physiotherapy, dietitian counselling, fundoscopy, dental care, etc.

2. Can you explain how these services have been helpful or not helpful in managing your condition?

Probes: Are services complete, timely, effective? What is missing or could be improved?

Affordability

3. Are there any charges or fees when you use healthcare services at this clinic?

Probes: Registration, lab tests, medication, specialist referrals.

4. How do you feel about these charges? Are they affordable or difficult for you to manage? Please explain.

Probes: Impact on household expenses, choices to skip care due to cost.

Accessibility

5. How do you usually travel to the clinic? What options are available to you?

Probes: Drive yourself, walk, family members, public transport.

6. Can you describe any challenges or ease you face in physically getting to the clinic?

Probes: Dependence on others, travel distance, transport costs, mobility issues.

Accommodation

7. Do the services at the clinic meet your personal needs and preferences?

Probes: Clinic hours, appointment scheduling, language used, gender of provider.

8. Have you ever needed services outside normal hours (evening, weekend)? Were they available? What did you do?

Acceptability

9. Have you ever felt that you were treated differently or unfairly by healthcare staff because of your personal background—such as age, gender, ethnicity, language, or religion? Please explain.

10. Do you feel comfortable receiving care from healthcare providers regardless of their age, gender, ethnicity, or religion? Why or why not?

Closing

11. Is there anything else you would like to share about your experiences in accessing diabetes care at this clinic?
